# Role of Virtual Ruler-Based Diameter Measurement in Endoscopic Therapy for Cirrhotic Esophageal Varices: A Retrospective Multicenter Study

**DOI:** 10.1155/cjgh/8823825

**Published:** 2024-11-29

**Authors:** Zhongliang Fang, Yuchuan Bai, Yudi Mao, Jing Jin, Qianqian Zhang, Yangchen Tang, Xiping Ding, Derun Kong

**Affiliations:** ^1^Department of Gastroenterology, The First Affiliated Hospital of Anhui Medical University, Hefei 230001, China; ^2^Department of Geriatrics and Gastroenterology, The First Affiliated Hospital of USTC, Hefei 230001, Anhui, China; ^3^Department of Geriatrics, Anhui Provincial Hospital (South District), Hefei 230001, Anhui, China

**Keywords:** esophageal varices, liver cirrhosis, rebleeding, virtual ruler

## Abstract

**Background:** Esophageal variceal (EV) diameter is a critical, independent risk factor for hemorrhage, and plays a key role in guiding choices of endoscopic treatment techniques. We developed a novel tool, the virtual ruler (VR), which offers increased precision and expediency in EV diameter (EVD) measurements. This study investigates the clinical value of VR for assessing EVD during the endoscopic treatment of cirrhotic EVs.

**Methods:** We performed a retrospective multicenter review of 345 cirrhotic patients with EVs who received endoscopic treatment. EVD was measured using VR, and several outcomes, including rebleeding rates, vascular eradication rates, mortality, and complication incidences, were compared in patients stratified by EVD as measured by both VR and endoscopists.

**Results:** There was moderate agreement between VR and endoscopist measurements of EVD (Kappa = 0.591, *p* < 0.001). In patients with EVD > 1 cm, the VR group had a lower rebleeding rate after endoscopic treatment compared to the endoscopist group (3.8% vs. 11.3%; *p*=0.048). No significant between-group differences in outcomes were noted in patients with EVD ≤ 1 cm. Additionally, comparisons of endoscopic variceal ligation and endoscopic injection sclerotherapy within the VR-based diameter groups showed no substantial differences in treatment efficacy or adverse events (*p* > 0.05).

**Conclusion:** Using VR to accurately measure EVD may help decrease endoscopist misjudgment of larger EVD values and may reduce postoperative rebleeding rates after endoscopic treatment. VR holds potential clinical significance in guiding endoscopic EV treatment.

**Trial Registration:** Clinical Trial Registry identifier: ChiCTR2200064028.

## 1. Introduction

Cirrhosis is the terminal phase of progressive liver fibrosis and is most commonly caused by chronic viral hepatitis, alcoholic liver disease, nonalcoholic fatty liver disease, autoimmune liver disorders, and parasitic infections [1, 2]. This condition is characterized by hepatic structural distortion and the development of regenerative nodules, leading to portal blood flow obstruction. This obstruction results in elevated portal venous pressure and the formation of collateral circulation. Key clinical manifestations include esophagogastric varices, hemorrhagic ruptures, hepatic encephalopathy, and ascites [3]. Variceal hemorrhages caused by esophageal varices (EVs) are gastrointestinal emergencies and are a major cause of mortality in cirrhotic patients. The mortality rate for each variceal hemorrhage episode can reach up to 20% [4], and there is a 70% risk of rebleeding within 1 year postepisode for survivors [5].

The degree of tension within variceal walls significantly influences the likelihood of rupture. According to Laplace's law of fluid mechanics, vessel wall tension is determined by the product of the difference between the internal and external vessel pressures and the vessel's diameter, divided by its wall thickness. This formula indicates that vessel wall tension results from both the pressure differential across the vessel wall and the vessel's diameter [6]. Increased internal pressure, accompanied by vessel dilation, reduces wall thickness and increases wall tension. When the vessel wall's elastic threshold is exceeded, rupture and significant hemorrhage are more likely to occur. Under consistent internal pressure, a larger diameter implies higher wall tension and, thus, a greater risk of rupture. The likelihood of variceal bleeding is independently correlated with the varices' diameter [7].

**Figure 1:**
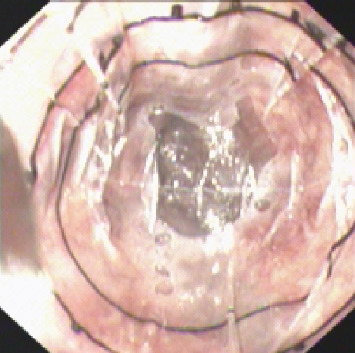
The diameter of the esophageal varices was gauged by the esophageal varix manometer, with each black tick mark separated by 0.5 cm. The largest esophageal varix in this figure has a diameter of 1 cm.

Endoscopic variceal ligation (EVL) and endoscopic injection sclerotherapy (EIS) are both effective methods for preventing and treating esophageal variceal bleeding [8]. Most clinical guidelines use factors such as EV size, the presence of red color signs, the patient's Child-Pugh score, and hepatic venous pressure gradients to guide treatment decisions [9, 10]. Variceal size is a crucial one of these factors, but relying solely on visual estimations for measurements is not very reliable and often requires advanced hardware [11, 12]. Therefore, our research team developed a novel technique called the virtual ruler (VR) for measuring esophageal variceal diameters (EVDs). In a previous study [13], we compared the VR to the gold-standard Esophageal Variceal Manometer (EVM). The balloon surface of the EVM features physical scales that are 0.5 cm each (Figure 1). Our previous study confirmed that there was a high degree of measurement consistency between EVM and VR. VR is also noninvasive and requires shorter measurement times.

Accurate EVD measurement is critical for predicting bleeding risk and formulating effective treatment plans, which is in turn, essential for ensuring patient safety and treatment efficacy [9–11]. This study uses retrospective case data to measure EVDs using VR and compares these measurements to those recorded by endoscopists. We also assess the impact of EVD measurements on the efficacy and safety of endoscopic interventions using both EVL and EIS techniques in cirrhotic patients. Thus, our overall aim is to evaluate the clinical utility of VR in assessing EVDs prior to endoscopic EV treatment in cirrhotic patients.

## 2. Materials and Methods

### 2.1. Patients

We enrolled 671 patients diagnosed with cirrhotic EVs who received endoscopic treatment between January 2017 and June 2023 at three medical centers: the First Affiliated Hospital of Anhui Medical University, the First Affiliated Hospital of the University of Science and Technology of China, and Anhui Provincial Hospital (South District). Case distribution was as follows: 441 patients at the First Affiliated Hospital of Anhui Medical University, 152 patients at the First Affiliated Hospital of the University of Science and Technology of China, and 78 patients at Anhui Provincial Hospital (South District).

#### 2.1.1. The Inclusion Criteria Were as Follows

Patients with (1) cirrhosis confirmed by hepatic biopsy, clinical assessment, or radiological evaluation, (2) EVs verified by endoscopy, (3) secondary prevention for EV bleeding, (4) EVs treated by EVL or EIS, and (5) a gastroscopy video or image that showed an endoscope equipped with a transparent cap with an inner diameter of 1 cm.

#### 2.1.2. The Exclusion Criteria Were as Follows

(1) Patients diagnosed with hematologic conditions or other significant organ-related comorbidities, (2) patients with gastroscopy videos or images that did not meet the specifications for VR measurement of blood vessel diameters (e.g., the front of the transparent cap fits snugly over the vessel and the vessel is fully visible at the top of the transparent cap), (3) patients with incomplete or missing data, and (4) patients who presented with isolated gastric varices (IGV) in the Sarin staging system.

Endoscopic procedures in this study were performed by experts with over 10 years of experience in endoscopic operations.

The study was conducted in compliance with the Declaration of Helsinki and Good Clinical Practice Guidelines, and received approval from the Ethics Committee of the First Affiliated Hospital of the University of Science and Technology of China (USTC) (Ethics Approval No. 2023KY-246). All patients provided informed consent for the endoscopic procedures. Because the study was retrospective and involved anonymized data from routine clinical procedures and tests, the need for specific research-related informed consent was waived.

### 2.2. Procedure for Using VR to Measure EVD

**Figure 2:**
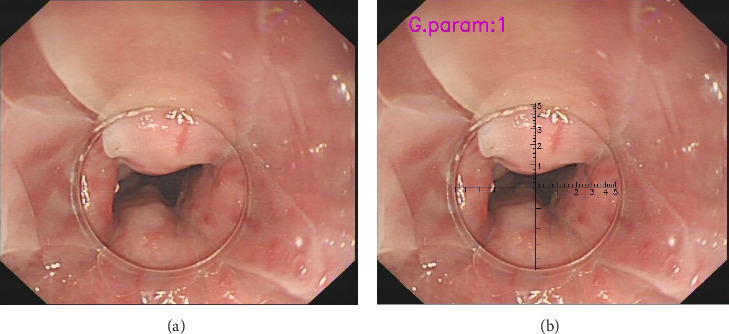
(a) Original image. (b) Utilizing the virtual ruler to ascertain the dimensions of esophageal varices. The diameter of the largest esophageal varix in this patient was found to be approximately 0.8 cm.

(1) Software Installation and Importing: Install the VR software and import the endoscopic video or image. Adjust the cursor on the VR ruler to align three of the four boundary points with the edge of the transparent cap, which has a 1-cm diameter. (2) Vessel Selection and Alignment: Select the blood vessel with the largest diameter for measurement. Ensure that the front end of the transparent cap is closely attached to the vessel and that the vessel is fully visible at the top of the cap. (3) Measurement: Use the scale and coordinates on the transparent cap's ruler to accurately measure the vessel's diameter (Figure 2). The VR features horizontal and vertical lengths of 1 cm, segmented into 10 grids of 1 mm each, and further divided into 0.2-mm subgrids. Our initial research thoroughly details the operational principles for VR [13].

### 2.3. Definitions

#### 2.3.1. Variceal Eradication

Variceal eradication was defined as the absence of blood flow within the esophageal wall, confirmed by endoscopic ultrasonography (EUS), as well as the absence of visible EVs during endoscopy [14].

#### 2.3.2. Rebleeding

Rebleeding was defined as an episode of bleeding from EVs with clinical signs such as hematemesis, melena, or a decrease in hemoglobin > 3 g/dL occurring more than 120 h post-treatment [15].

#### 2.3.3. EVs Forms

EVs were classified into three forms: F1 (straight and small-caliber), F2 (moderately enlarged and beady), and F3 (markedly enlarged, nodular, or tumor-shaped) [16].

#### 2.3.4. EVL

EVL involves using elastic bands to ligate the bases of varicose veins under endoscopic guidance. This procedure causes ischemia, necrosis, and detachment of the varicose veins, which occludes them. It effectively controls active variceal bleeding and eliminates varices [17]. The procedural steps include preoperative preparation, general anesthesia, attachment of the ligation device to the endoscope, deployment of the ligation band, postprocedural observation, and subsequent postoperative care.

#### 2.3.5. EIS

EIS involves injecting sclerosing agents into the most prominent EV location in the lower esophagus under endoscopic guidance. This injection induces aseptic inflammation by damaging the endothelial cells, leading to granulation tissue formation and subsequent fibrosis, which ultimately results in vein occlusion [18]. The procedural steps include preoperative preparation, general anesthesia, endoscopic localization of the treatment site, injection of the sclerosant, and postoperative care.

### 2.4. Data Collection and Follow-Up

Data collection included demographic characteristics, endoscopy data, variceal eradication rates, rebleeding rates, complication rates, and other relevant information. Follow-up endoscopy was performed 1 month postoperatively to evaluate EV status, and the final follow-up was conducted on July 30, 2023.

### 2.5. Statistical Analysis

Quantitative data are reported as means ± standard deviations (SD), with group comparisons conducted using independent samples *t*-tests. Categorical variables are expressed as percentages and analyzed using chi-square tests. When the chi-square test conditions were not met, Fisher's exact probability tests were used. Statistical significance was set at a *p* value of less than 0.05. Kappa tests were used to assess the consistency between VR and endoscopist-conducted EVD measurements. All statistical analyses were conducted using SPSS (version 23; IBM Corp.).

## 3. Results

### 3.1. Baseline Patient Characteristics

**Figure 3:**
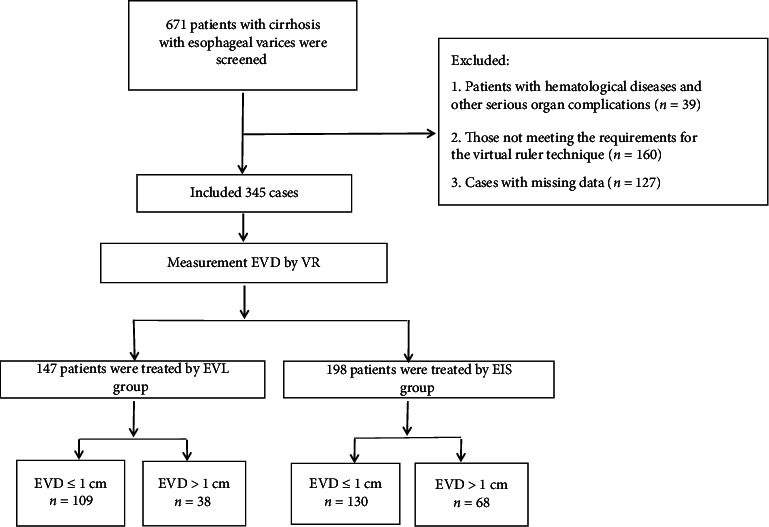
Clinical trial flowchart. EIS, endoscopic injection sclerotherapy; EVD, esophageal varices diameter; EVL, endoscopic variceal ligation; VR, virtual ruler.

A total of 671 patients with cirrhosis and EVs were treated across three medical centers in this study. As shown in Figure 3, 326 patients were excluded because they did not meet the inclusion criteria, leaving 345 patients in the final analysis. Among the included patients, 147 were in the EVL group, 109 of whom had EVDs ≤ 1 cm and 38 of whom had EVDs > 1 cm. The EIS group was comprised of 198 patients, 130 of whom had EVDs ≤ 1 cm and 68 of whom had EVDs > 1 cm. The study cohort consisted of 243 males (70.4%) and 102 females (29.6%), with an average age of 53.36 ± 11.55 years. Hepatitis B viral infection was the primary cause of cirrhosis and in this study (*n* = 174, 50.4%). Based on the Child-Pugh scoring system, 212 patients (61.4%) were classified as grade A, and 122 patients (35.4%) were classified as grade B. All patients received nonselective beta-blockers (NSBBs) for bleeding prevention. Varix typing revealed the following: EV (*n* = 181, 52.5%) and EV + GOV1 (*n* = 140, 40.6%). Varix grading was primarily F2 (*n* = 238, 69%) and F3 (*n* = 92, 26.7%). During the follow-up period, two patients died because of nonhemorrhagic factors. The clinical characteristics of our cohort are summarized in Table 1.

### 3.2. Evaluation of Consistency Between VR and Endoscopist-Measured EVD

In the present study, endoscopist-measured EVDs were ≤1 cm in 265 cases and > 1 cm in 80 cases. In contrast, the VR-measured EVDs were ≤ 1 cm in 239 cases and > 1 cm in 106 cases. Kappa consistency tests produced a value of 0.591 (*p* < 0.001), demonstrating moderate agreement between the two measurement methods. Misjudgment of EVD size was observed in 56 patients, including 41 patients with an endoscopist-measured EVD ≤ 1 cm who had a VR-measured EVD > 1 cm, and 15 patients with an endoscopist-measured EVD > 1 cm who had a VR-measured EVD ≤ 1 cm. Detailed data are available in Table 2.

### 3.3. Correlations Between EVD Measured by VR and Endoscopists and the Efficacy and Safety of Endoscopic Treatments (EVD ≤ 1 cm)

Among patients with EVDs ≤ 1 cm, the VR group had higher vascular eradication rates than the endoscopist group, along with lower rebleeding and mortality rates. However, these differences did not achieve statistical significance (*p* > 0.05). Rebleeding occurred in 34 (14.2%) patients in the VR group and 29 (10.9%) in the endoscopist group. Detailed data are available in Table 3.

### 3.4. Correlations Between EVD Measured by VR and Endoscopists and the Efficacy and Safety of Endoscopic Treatments (EVD > 1 cm)

In patients with EVDs > 1 cm, no significant between-group differences were observed in variceal eradication rates, mortality rates, or complication rates (*p* > 0.05). Conversely, the VR group had a lower rebleeding rate compared to the endoscopist group, and this difference was statistically significant (*p* < 0.05). Rebleeding occurred in four cases (3.8%) in the VR group and nine cases (11.3%) in the endoscopist group. Detailed data are available in Table 4.

### 3.5. Comparisons of the Efficacy and Safety of EVL and EIS in Patients With EVD ≤ 1 cm Based on VR

In a group with VR-measured EVD of ≤ 1 cm, there were no significant differences in rebleeding rates, variceal eradication rates, mortality rates, or complication rates between EVL and EIS (*p* > 0.05). Rebleeding occurred in 13 cases (11.9%) in the EVL group and 21 cases (16.2%) in the EIS group. Detailed data are presented in Table 5.

### 3.6. Comparisons of the Efficacy and Safety of EVL and EIS in Patients With EVD > 1 cm Based on VR

In a group with VR-measured EVD of > 1 cm, there were no significant differences in rebleeding rates, variceal eradication rates, mortality rates, or complication rates between EVL and EIS (*p* > 0.05). Rebleeding occurred in two cases (5.3%) in the EVL group and two cases (2.9%) in the EIS group. Detailed data are available in Table 6.

## 4. Discussion

Existing methods for measuring blood vessel diameters include visual inspection measurement, VR measurement, EVM measurement, and CHESS ruler measurement. Comparisons between VR and endoscopist-led visual inspection suggest that VR can provide a reference ruler. In contrast to the VR, the CHESS ruler is a physical ruler, needs to be unfolded in the esophageal lumen, and has a maximum diameter of 1 cm [19]. Further research is also required to ensure CHESS' safety and applicability. EVM is primarily used to simultaneously gauge EV pressure and circumferences. However, EVM is seldom used in clinical settings. Our previous study's findings unequivocally validated the precision of the VR technique in measuring EVD. This study also demonstrated that VR is a more time-efficient and noninvasive method for measuring EVs than EVM, reinforcing its clinical utility [13].

The artificial intelligence-based software underlying our VR includes algorithms such as Gaussian filters, Canny edge detectors, and Hough circles. As the VR approaches the target varicose vein, it detects the discontinuous arc of the hyaline cap and establishes a Cartesian coordinate system at the center of the circle which corresponds to the arc. The VR then uses the established coordinate system to determine the diameter of the varicose vein by comparing it to a calibrated scale obtained from barrel deformation experiments.

In this study, we found moderate agreement between the VR and endoscopists in categorizing patients into the EVD ≤ 1 cm and EVD > 1 cm groups. Notably, EVD size misjudgment occurred in 56 patients, including 41 patients with an endoscopist-measured EVD ≤ 1 cm who had a VR-measured EVD > 1 cm, and 15 patients with an endoscopist-measured EVD > 1 cm who had a VR-measured EVD ≤ 1 cm. The VR-measured EVD exhibited greater accuracy than endoscopists. Accurately determining EV size can influence the choice of treatment modality and treatment site, and VR can identify more EVs with diameters > 1 cm, reducing misclassification rates. Precise EVD measurement may also improve treatment outcomes and reduce complication rates.

In our study, we had 106 patients with VR-defined EVDs that were > 1 cm. We found that these VR measurements were closer to the true vessel diameters than endoscopist-based measurements, and four cases (3.8%) experienced rebleeding. In contrast, the number of patients classified by endoscopists as having EVDs > 1 cm was only 80, and there were nine cases of rebleeding (11.3%). This difference suggests that misjudgment by the human eye can lead to a higher rate of postoperative hemorrhage in these cases (e.g., in cases physicians misclassified as having EVDs > 1 cm). Thus, using VR to preoperatively determine cases with EVDs > 1 cm may decrease the likelihood of postoperative rebleeding.

In this study, the rebleeding rate was 14.2% amongst the 239 patients with EVD (VR) ≤ 1 cm (Table 3), and the rebleeding rate was 3.8% amongst the 106 patients with EVD (VR) > 1 cm (Table 4). These findings suggest that there is a higher rate of postoperative rebleeding in smaller vessels. This may be because EIS for smaller vessels is difficult, that is with small vessels, it is easier to accidentally inject the sclerosing agent into the submucosal layer rather than the varices, which can result in esophageal ulceration-induced rebleeding. As shown in Table 5, the rebleeding rate amongst patients with EVDs (VR) ≤ 1 cm was 16.2%, while the rebleeding rate amongst patients with EVDs (VR) > 1 cm was 2.9%. However, some of the included cases represented patients who had received multiple treatments and may have had residual small vessels or scar formation, which also increased the difficulty of EVL operation rebleeding risk. This could explain why the EVDs (VR) ≤ 1 cm rebleeding rate was 11.9% in Table 5, while the rebleeding rate after EVL in patients with EVDs (VR) > 1 cm was 5.3% in Table 6.

We found no significant differences between the VR group and the endoscopist group regarding efficacy and safety outcomes for patients with EVD ≤ 1 cm. This is likely due to the high accuracy of endoscopists in evaluating smaller EVs and the proven effectiveness of endoscopic therapy for smaller EVs.

Our group has previously examined the relationship between rebleeding rates and various potential risk factors. Xiao et al. [20] demonstrated that Child-Pugh classification, ALB levels, PT, EVD (VR value), and red flag signs were closely linked to rebleeding after endoscopic EV treatment, with the highest rate of EV rebleeding noted in the group with EVDs > 1 cm. Cao et al. [21] identified risk factors for early rebleeding after EVL, including high TB, low Alb, high PT, PVT, HCC, elevated Child-Pugh scores, Child-Pugh class C, high MELD score, Japanese varicose vein class F3, EV diameter, and the number of ligature rings. Consequently, the present study did not further elaborate on the rebleeding rate in relation to associated risk factors.

EVL is generally recommended for the prevention and treatment of EVs, as supported by numerous guidelines [10, 22]. Evidence-based medicine provides moderate-certainty evidence favoring EVL over EIS for the secondary prevention of variceal bleeding [23]. However, EIS is more widely used because of its effectiveness in treating deep varicose veins and collateral vessels, as well as its lower recurrence rate compared to EVL [24]. EVL is typically employed for EVs with diameters < 2 cm due to device limitations, while EIS can be used for larger varices because it is not constrained by EV size. Our findings indicate that, for patients with VR-measured EVD ≤ 1 cm and > 1 cm, there were no statistically significant differences in rebleeding rates, variceal eradication rates, mortality rates, or complication rates between the EVL and EIS groups. Comprehensive analysis suggests that the EVD of the EVs selected for EVL should not be larger than 1 cm.

This study has several limitations. First, as a retrospective study, it is subject to several potential biases, such as selection bias, recall bias, limitations in causal inference, and difficulties in controlling for confounders. Second, the relatively small sample size may have reduced statistical power, resulted in less stable outcomes, and increased the potential for bias. Third, due to the current limitations of the VR technology, only EVDs ≤ 1 cm could be measured accurately. Measurements for EVDs larger than 1 cm were categorized as > 1 cm, preventing further segmentation and subgroup analysis for larger diameters. Enhancing VR's capacity to accurately quantify EVDs and conducting extensive prospective studies are necessary to fully assess the clinical utility of VR.

In conclusion, this study represents a pioneering application of VR in the clinical assessment of EVs. Using VR to accurately measure EVDs may help decrease instances of endoscopists misjudging larger EVD values and lower the rate of postoperative rebleeding after endoscopic treatments. The VR thus has potential clinical significance in guiding the endoscopic treatment of EVs.

## Figures and Tables

**Table 1:** Baseline patient characteristics.

Characteristic	Value *n* (%)/mean ± SD
Age, years	53.36 ± 11.55
Sex	
Male	243 (70.4)
Female	102 (29.6)
Etiology of cirrhosis	
Hepatitis B virus	174 (50.4)
Alcohol abuse	28 (8.1)
Autoimmunity	36 (10.4)
Schistosomiasis	8 (2.3)
Combined	23 (6.7)
Others	76 (22)
Splenectomy	45 (13.0)
TIPs	11 (3.2)
Hepatic encephalopathy	4 (1.2)
Concomitant hepatocellular carcinoma	37 (10.7)
Portal vein thrombosis	88 (25.5)
Serum albumin, g/L	33.94 ± 4.96
Serum bilirubin, *μ*mol/L	23.22 ± 15.5
Hemoglobin, g/L	83.2 ± 23.05
PLT,10^9^/L	90.51 ± 75.64
AST, IU/L	54.67 ± 168.44
ALT, IU/L	52.05 ± 242.94
Creatinine, *μ*mol/L	65.33 ± 34.76
PT, S	15.15 ± 2.89
PT-INR	1.22 ± 0.26
Child-Pugh score	
A	212 (61.4)
B	122 (35.4)
C	11 (3.2)
EVs variceal form	
F1	15 (4.3)
F2	238 (69)
F3	92 (26.7)
Red color signs	322 (93.3)
EVD (Endo)	
≤ 1 cm	265 (76.8)
> 1 cm	80 (23.2)
EVD (VR)	
≤ 1 cm	239 (69.3)
> 1 cm	106 (30.7)

Abbreviations: ALT, alanine transaminase; AST, aspartate transaminase; EIS, endoscopic injection sclerotherapy; Endo, endoscopists; EVD, the diameter of esophageal varices; EVL, endoscopic variceal ligation; EVs, esophageal varices; PLT, platelet; PT-INR, prothrombin time international normalized ratio; SD, standard deviation; TIPs, transjugular intrahepatic portosystemic.

**Table 2:** Consistency evaluation of EVD measured by VR and endoscopists.

EVD (endo)	EVD (VR)		Total	Kappa	*p*
	≤ 1 cm	> 1 cm			
≤ 1 cm	224	41	265	0.591	< 0.001
> 1 cm	15	65	80		
Total	239	106	345		

Abbreviations: Endo, endoscopists; EVD, the diameter of esophageal varices; VR, virtual ruler.

**Table 3:** Comparison of rebleeding rate, variceal eradication rate, mortality rate, and incidence of complications between VR and endoscopists in patients with EVD ≤ 1 cm.

Variables	*N* VR/Endo	VR *N* (%)	Endo *N* (%)	*χ* ^2^	*p*
Rebleeding	239/265	34 (14.2)	29 (10.9)	1.238	0.266
Variceal eradication	239/263	55 (23)	57 (21.7)	0.130	0.719
Mortality	239/265	0 (0)	2 (0.8)	—	0.500
Complications					
Fever	239/265	1 (0.4)	4 (1.5)	1.523	0.217
Retrosternal chest discomfort	239/265	46 (19.2)	49 (18.5)	0.047	0.828
Abdominal bloating	239/265	8 (3.3)	10 (3.8)	0.066	0.797
Nausea	239/265	4 (1.7)	4 (1.5)	0.000	1.000
Vomiting	239/265	3 (1.3)	3 (1.1)	0.000	1.000
Total	239/265	59 (24.7)	67 (25.3)	0.024	0.877

Abbreviations: Endo, endoscopists; EVD, the diameter of esophageal varices; VR, virtual ruler.

**Table 4:** Comparison of rebleeding rate, variceal eradication rate, mortality rate, and incidence of complications between VR and endoscopists in patients with EVD > 1 cm.

Variables	*N* (VR/Endo)	VR *N* (%)	Endo *N* (%)	*χ* ^2^	*p*
Rebleeding	106/80	4 (3.8)	9 (11.3)	3.920	0.048
Variceal eradication	104/80	14 (13.5)	12 (15)	0.088	0.766
Mortality	106/80	2 (1.9)	0 (0)	—	0.507
Complications					
Fever	106/80	5 (4.7)	2 (2.5)	0.158	0.691
Retrosternal chest discomfort	106/80	19 (17.9)	16 (20)	0.129	0.720
Abdominal bloating	106/80	6 (5.7)	4 (5)	0.000	1.000
Nausea	106/80	0 (0)	0 (0)	—	—
Vomiting	106/80	0 (0)	0 (0)	—	—
Total	106/80	30 (28.3)	22 (27.5)	0.015	0.904

Abbreviations: Endo, endoscopists; EVD, the diameter of esophageal varices; VR, virtual ruler.

**Table 5:** Comparison of rebleeding rate, variceal eradication rate, mortality rate, and incidence of complications for EVL versus EIS in patients with EVD ≤ 1 cm as measured by VR.

Variables	*N* (EVL/EIS)	EVL *N* (%)	EIS *N* (%)	*χ* ^2^	*p*
Rebleeding	109/130	13 (11.9)	21 (16.2)	0.868	0.351
Variceal eradication	109/130	22 (20.2)	33 (25.4)	0.905	0.341
Mortality	109/130	0 (0)	0 (0)	—	—
Complications					
Fever	109/130	1 (0.9)	0 (0)	—	0.456
Retrosternal chest discomfort	109/130	24 (22)	22 (16.9)	0.990	0.320
Abdominal bloating	109/130	4 (3.7)	4 (3.1)	0.000	1.000
Nausea	109/130	3 (2.8)	1 (0.8)	0.468	0.494
Vomiting	109/130	3 (2.8)	0 (0)	1.743	0.187
Total	109/130	32 (29.4)	27 (20.8)	2.352	0.125

Abbreviations: EIS, endoscopic injection sclerotherapy; EVD, esophageal varices diameter; EVL, endoscopic variceal ligation; VR, virtual ruler.

**Table 6:** Comparison of rebleeding rate, variceal eradication rate, mortality rate, and incidence of complications for EVL versus EIS in patients with EVD > 1 cm as measured by VR.

Variables	*N* (EVL/EIS)	EVL *N* (%)	EIS *N* (%)	*χ* ^2^	*p*
Rebleeding	38/68	2 (5.3)	2 (2.9)	0.005	0.944
Variceal eradication	37/67	2 (5.4)	12 (17.9)	2.216	0.137
Mortality	38/68	1 (2.6)	1 (1.5)	—	1.000
Complications					
Fever	38/68	3 (7.9)	2 (2.9)	0.457	0.499
Retrosternal chest discomfort	38/68	7 (18.4)	12 (17.6)	0.010	0.921
Abdominal bloating	38/68	4 (10.5)	2 (2.9)	1.398	0.237
Nausea	38/68	0 (0)	0 (0)	—	—
Vomiting	38/68	0 (0)	0 (0)	—	—
Total	38/68	14 (36.8)	16 (23.5)	2.129	0.145

Abbreviations: EIS, endoscopic injection sclerotherapy; EVD, esophageal varices diameter; EVL, endoscopic variceal ligation; VR, virtual ruler.

## Data Availability

The data used to support the findings of the study are available from the corresponding author on reasonable request.
